# Comparisons of the surface micromotions of cementless femoral prosthesis in the horizontal and vertical levels: a network analysis of biomechanical studies

**DOI:** 10.1186/s13018-020-01794-4

**Published:** 2020-07-31

**Authors:** Bomin Wang, Qinghu Li, Jinlei Dong, Dongsheng Zhou, Fanxiao Liu

**Affiliations:** Department of Orthopaedics, Shandong Provincial Hospital Affiliated to Shandong First Medical University, No. 324, Road Jing Wu Wei Qi, Jinan, 250021 Shandong Province China

**Keywords:** Biomechanics, Femoral stem, Micromotion, Network meta-analysis

## Abstract

**Background:**

Numerous quantitatively biomechanical studies measuring the fixation stability of femoral stem using micromotions at the bone-implant interfaces in different directions and levels remain inconclusive. This network meta-analysis performed systematically aims to explore the rank probability of micromotions at the bone-implant interfaces based on biomechanical data from studies published.

**Methods:**

Two electronic databases, PubMed/MEDLINE and Embase, were utilized to retrieve biomechanical studies providing the data of micromotions at the bone-stem interfaces. After screening and diluting out, the studies that met inclusion criteria will be utilized for statistical analysis. In order to contrast the stability of commonness and differences of the different parts of the femoral stem, the horizontal and vertical comparison of micromotions at the bone-implant interfaces were conducted using the pooled evaluation indexes including the mean difference (MD) and the surface under the cumulative ranking (SUCRA) curve, while inconsistency analysis, sensitivity analysis, subgroup analyses, and publication bias were performed for the stability evaluation of outcomes.

**Results:**

Screening determined that 20 studies involving a total of 249 samples were deemed viable for inclusion in the network meta-analysis. Tip point registered the highest micromotions of 13 measurement points. In the horizontal level, the arrangements of 4 measurement points at the proximal (P1–P4), middle (P5–P8) and distal part of the stem (P9–P12) were P1 = P2 = P3 = P4, P7 > P8 > P6 = P5 and P10 ≥ P12 = P9 = P11, respectively. In the vertical level, the arrangements of 3 measurement points at the anterior, posterior, medial, and lateral directions was P9 > P5 = P1, P10 > P6 > P2, P11 > P7 > P3, and P12 > P8 > P4, respectively.

**Conclusion:**

The network meta-analysis seems to reveal that the distal part of the femoral stem is easier to register higher micromotion, and tip point of femoral stem registers the highest micromotions.

## Introduction

Currently, biomechanical tests are usually performed on synthetic or cadaveric specimens to evaluate the fixation stability of orthopedic implants at the hip, shoulder, and radius and for tendon repairs using bone anchoring devices [1–6]. In vitro investigations represent a decisive part in preclinical testing of cementless implants [7]. Though cemented as well as cementless total hip arthroplasty (THA) demonstrated a satisfactory survival rate (90%, 95%, and 97% at 5, 10, and 15 years follow-up, respectively) [8], aseptic loosening of artificial joint, resulting from the absence of primary stability, wear, and periprosthetical osteolysis as a result of the implant-specific bone remodeling, is one of the most common long-term complications in clinical, which limits the prospective efficacy and service life of prostheses [8].

With the development of the surgical technique and improvement of the material and design of the implant, these complications will reduce, but a further follow-up is still necessary. In contrast to cemented implants that achieve ultimate stability directly after implantation, cementless implants rely on their mechanically firm fit and lock between implant and bone (primary stability) and consequently biological osseous integration of the implant (secondary stability) [9], which has become increasingly popular over the past few years [10].

In total hip replacement, key point of prophylaxis for aseptic loosening was prophylaxis for periprosthetic osteolysis which was due to particulate wear debris induced-biological reaction in histiocyte surrounding the prosthesis. The rationale for the measurement micromotion between bone and implant is based on animal studies that demonstrated that excess movement (150 μm or more) can result in the failure of osseous integration of cementless implants [11, 12]. Therefore, the importance where the aseptic looseness occurs firstly cannot be overstressed, since they are essential for implant design.

To date, various methods have been developed and introduced to register micromotions [13–15]. Biomechanical investigation, as the most frequently used survey method, is applied to measure the relative micromotions at the bone-implant interface in total hip replacement arthroplasty (THA) based on two different kinds of specimens, fresh frozen cadaver bones and artificial composite femurs [16].

In fact, multiple biomechanical studies [13–15, 17–33] have reported to measure the relative micromotions at the bone-implant interface in the THA, with different length of femoral stem, sizes of femurs, loading scenarios, different specimens, implant types and sizes, and implant designs. Due to various loading scenarios along with numerous measurement devices and locations, comparisons of the micromotions of different research laboratories are very difficult [16].

To the best of our knowledge, results of the micromotions between bone and implant in the same specimen at different points on biomechanical test remain unknown, while various biomechanical studies have been performed which attempted to quantitatively measuring of the micromotions of femoral stem prosthesis at different levels. As such, the aims of the present study were to pool all available scientific published biomechanical material using strict inclusion and exclusion criteria followed by meta-analysis, compare the implant bone interface micromotions at different points of femoral stem, and explore the mechanism of aseptic loosening of artificial hip joint.

## Materials and methods

This investigation was conducted based on “the Preferred Reporting Items for Systematic Reviews and Meta-Analyses (PRISMA)” statement [34].

### Inclusion and exclusion criteria

The inclusion criteria were (1) biomechanical studies, (2) studies evaluating the micromotions between femoral stem and bone, and (3) studies providing available data to calculate evaluation index including mean and standard deviation of each point.

The exclusion criteria were (1) correspondence, commentary, reviews, editorials, systematic review, meta-analysis, and other non-original studies; (2) congress proceedings; (3) animal experiments; (4) finite analysis, and (5) biomechanical studies providing no data to calculate the evaluation index.

### Data sources and search strategy

The following search terms were adopted in the network meta-analysis: “femoral” [All Fields], “femur” [All Fields], “femora” [All Fields], “total hip arthroplasty” [All Fields], “THA” [All Fields], “hip replacement” [All Fields], or “hemi-hip replacement” [All Fields] AND “biomechanics” [All Fields], “biomechanical” [All Fields], “biology mechanics” [All Fields], “finite element analysis” [All Fields], “finite element” [All Fields] or “FEA” [All Fields].

To be fully searched, potential relevant articles came from two sources. Firstly, two electronic databases including PubMed/MEDLINE and Embase were searched for all potential relevant articles without any language and search restrictions. Subsequently, all bibliographies of pertinent articles (included studies, reviews, systematic reviews, and meta-analyses) were further screened manually to retrieve additional studies that were omitted in the initial search.

### Data extraction and quality assessment

**Fig. 1:**
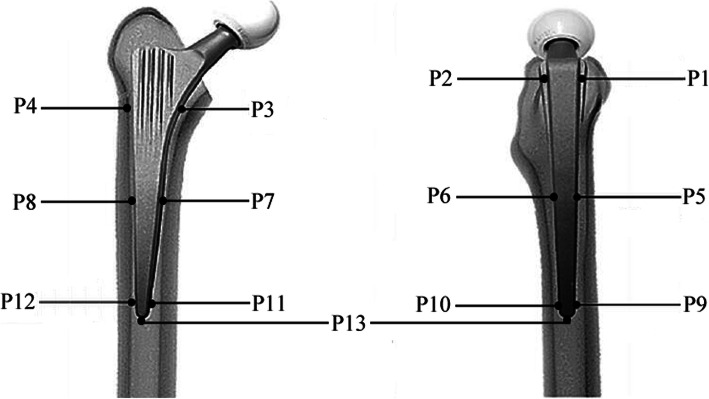
The schematic diagram of measuring micromotions of 13 measurement points at the bone-stem interfaces

The following information was deemed appropriate for inclusion: (1) basic information including the first author’s surname, year of publication, and country of origin; (2) specimen information including the number of femurs and donors, age and gender of donors, and femoral osteotomy based on cadaver specimens; (3) composite femur information including characteristics and femoral osteotomy based on composite specimens; (4) implant information including brand, coating, characteristics, material testing machine, and test jig; (5) loading information including action and anatomical features of simulation, load force, femur orientation, and test cycles; and (6) micromotion of 13 measurement points (P1, P5, and P9 at the anterior direction in the proximal, middle, and distal part of femoral stem; P2, P6, and P10 at the posterior direction in the proximal, middle, and distal part of femoral stem, respectively; P3, P7, and P11 at the medial direction in the proximal, middle, and distal part of femoral stem; P4, P8, and P12 at the lateral direction in the proximal, middle, and distal part of femoral stem; P13 at the tip of femoral stem) (Fig. 1).

A modified version of the Strengthening the Reporting of Observational studies in Epidemiology (STROBE) [35] checklist, a quality assessment tool, was used to estimate the methodological quality of the included studies, which considered 10 items including aim description, dependent variables description, interventions description, load/speed order randomized, setting description, date collection description, data analysis description, statistical description, drop outs, and point estimate/variability. Modifications were made to the STROBE checklist to only identify reporting criteria essential for the judgment of risk of bias, judgment of external generalizability of results, and replicability of the study’s methods.

### Statistical analysis

To reduce potential bias, two blinded and independently working investigators (LFX and DJL) searched two electrical databases to screen and identify the targeted articles using the aforementioned keyword and inclusion criteria, extracted the useful data, and assessed the quality of included studies using STROBE [35]. Any discrepancies were resolved by consensus from discussion. Inter-reviewer agreement was calculated at title, abstract, and full-text stage, including the quality assessment of the included studies stage, and quality was assessed with a Kappa (*κ*) statistic. Agreement was categorized a priori as follows: 0.20 or less, poor agreement; 0.21 to 0.40, fair agreement; 0.41 to 0.60, moderate agreement; 0.61 to 0.80, substantial agreement; and 0.81 to 0.99, nearly perfect agreement [36].

R (R-v-3.4.3), rjags, and package of gemtc was used to perform the Bayesian network meta-analysis [37, 38]. Inconsistency test and homogeneity analysis were performed using node analysis method. According to the Cochrane handbook, heterogeneity cross studies was assessed using *Q* test (*P* < 0.05 indicating the presence of heterogeneity) and *I*^2^ test (0–40%, heterogeneity might not be present; 30–50%, moderate heterogeneity; 50–90%, substantial heterogeneity; and 75–100%, considerable heterogeneity) [39, 40]. The sensitivity analysis was performed by comparing the differences of two effect models including fixed-effect and random-effect model. The clinical outcome indicators were evaluated by the mean difference (MD) for continuous outcomes. The difference in mean values of displacement outcome was compared and had undergone 13 measurement points. The surface under the cumulative ranking (SUCRA) curve was conducted to discuss rank probability [37, 38]. *P* < 0.05 was accepted as indicative of statistical significance.

## Results

### Selection process, study characteristic, and quality assessment

**Fig. 2:**
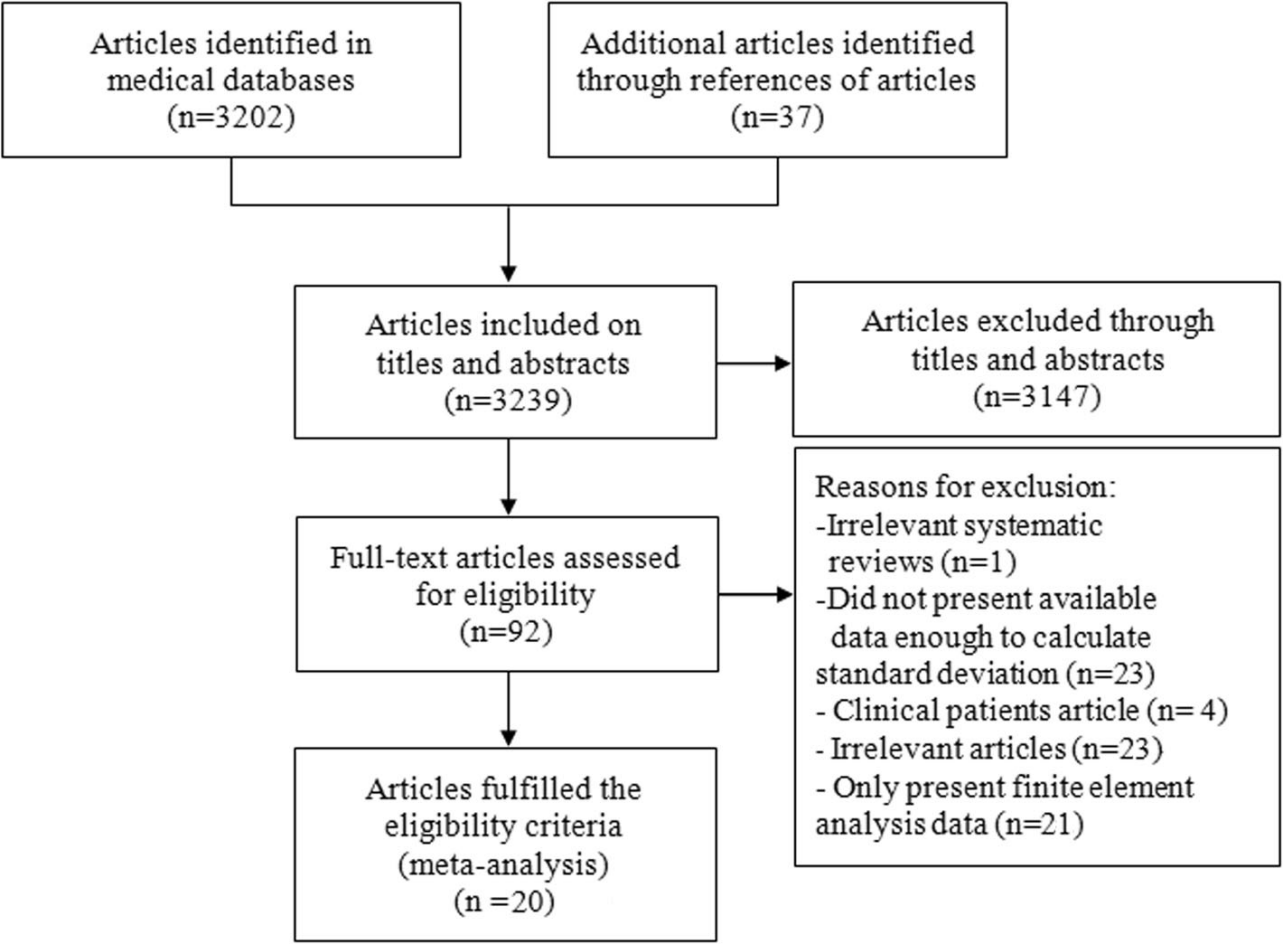
Flow chart for the search selection of all included biomechanical studies

The detailed article search and study selection process is listed in Fig. 2. A total of 3202 articles were retrieved after the initial search of the chosen electronic databases, with 37 additional articles being localized that originated from the references lists from the relevant studies scanned for in the databases. Of the 3239 articles scanned, 92 could be the target trails. Seventy-four articles were excluded from the selection process after further scanning where 23 articles were irrelevant publications, 21 articles only presenting finite element analysis data, one article that was a review, and 20 articles lacked sufficient data to be feasible for inclusion in the meta-analyses to calculate statistical index. After careful selection, eventually, 20 articles involving 249 specimens (148 composites and 101 cadaver femurs) were used for the network meta-analysis (website links enabling direct access to the abstract of included studies in Appendix 1). Articles with sample sizes ranging from 1 to 20 specimens were published from 2000 to 2017. Substantial agreement among the reviewers was achieved at each stage: title (*κ* = 0.73, 95% CI 0.69 to 0.78), abstract (*κ* = 0.87, 95% CI 0.82 to 0.93), and full-text (*κ* = 0.85, 95% CI 0.75 to 0.95) as well as the substantial agreement regarding the quality assessment of the included studies (*κ* = 0.83, 95% CI 0.72 to 0.89). The main characteristics of the studies utilized in the network meta-analysis are represented in Tables 1, 2, 3, and 4. The detail methodological quality of the studies utilized in the network meta-analysis is represented in Table 5.

**Table 1:** Specimen and implant information of included studies with cadaver specimens

Author, year	Country	Specimen information			The Position of femoral osteotomy	Subjects	Implant information		
		Femurs	Donors	Mean age (range)			Brand	Coating	Characteristics
Gotze 2002 [20]	Germany	7R, 7 L	F 1, M 6	57.0 (39–78)	37 cm proximal to the condyles	7	Adaptiva	Customized reamer	12/14 taper; TiAl6V4 alloy
						7	Alloclassic SL stem	Grit-blasted surface	12/14 taper; TiAl6V4 alloy
Klestil 2006 [21]	Austria	7R, 7 L	4F¸ 3 M	74.4 (63–88)	Femora was cut to a length of 25 cm	10	Endo SL stem	Ø 50 mm, CoCr head	NR
						10	FMT stem	Ø 28 mm, CoCr head	
Abdul-Kadir 2008 [17]	USA	4	NR	NR	25 cm below the lesser trochanter	4	Alloclassic hip stem	Full with a grit-blasted surface	NR
Pettersen 2009 [23]	Norway	6R, 6 L	F 3, M 3	61.0 (49–74)	25 cm below the greater trochanter	10	Summit TM straight stem	Dual coated with porocoat and HA as well as grit-blasted distally	Straight titanium stem
Østbyhaug 2010 [22]	Norway	10R, 10 L	F 1, M 9	51.6 (27–68)	25 cm below the greater trochanter	10	ABG-I anatomical stem	Proximal 1/3 of stem coated with 50 μm HA; Distal 2/3 is non-polished	Titanium alloy Ti6Al4V
						10	Unique customized stem	Proximal 2/3 coated with 50 μm HA; distal 1/3 is polished and downscaled	
Wik 2011 [24]	Norway	10R, 10 L	F 3, M 7	57.8 (44–71)	25 cm below the greater trochanter	10	Summit TM straight stem	Dual coated with porocoat and HA as well as grit-blasted distally	Straight titanium stem
Bieger 2012 [18]	Germany	11R, 11 L	4F, 8 M	60.4 (31–78)	37 cm below the greater trochanter	6	Fitmore short stem	Proximal 1/2 coated with plasma	Tapered design; trapezoidal cross-section
						10	CLS Spotorno stem	Full grit-blasted surface	Double-tapered design; straight collarless; rectangular cross-section; Size 13
						6	Mayo conservative short stem	Aluminum oxide roughed surface	Wedge-shaped; double-tapered design
Bieger 2013 [19]	Germany	9R, 9 L	7F, 2 M	53.5 (25–5x)	37 cm below the greater trochanter	9	CBC straight stem	Full with a corundum-blasted surface; Proximal part with prism-shaped ribs	Titanium alloy; double-tapered design
						9	Optimys short stem	Proximal part coated with titanium plasma-sprayed surface; distal tip is polished	Titanium alloy; triple-trapered design
Østbyhaug 2013 [15]	Norway	6R, 6 L	F 1, M 5	52.4 (48–61)	25 cm below the greater trochanter	10	ABG-I anatomical stem	Proximal 1/3 coated with 50 μm HA; distal 2/3 is non-polished	Titanium alloy Ti6Al4V
						10	-10		
						10	-20		
						10	-30		
						10	-40		
						10	-50		
Bieger 2016 [19]	Germany	6R, 6 L	3F, 3 M	38.0 (19–52)	37 cm below the greater trochanter	6	CBH straight stem	Proximal wing-shaped shoulder; rough-blasted	Zweymüller-type stem; rectangular cross-section; tapered design
						6	CBH bone-preserving stem	Rough-blasted; proximal lateral was reduced; the tip was flattened	Titanium alloy; rectangular cross-section; tapered design

*HA* hydroxyapatite, *F* female, *M* male, *L* left, *R* right, *NR* not reported

**Table 2:** Loading information of included studies with cadaver specimens

Author, year	Material testing machine	Testing jig	Loading information				
			Action simulated	Anatomical features simulated	Load force	Femur orientation	Test cycles
Gotze 2002 [20]	Zwick, Typ 1454, Einsingen, Germany	6 LVDTs	NR	NR	Vertical force 200–2000 N, 0.5 Hz	8° valgus, 6° flexion	1
Klestil 2006 [21]	Bionics 851.2, MTS	NR	NR	Acetabulum	50–2100 N; frequency (2 Hz)	10° flexion, 10° adduction	50000
Abdul-Kadir 2008 [17]	Instron 5565, Instron Corp., Canton, MA, USA	2 LVDTs	NR	NR	5000 N load cell with at a rate of 1 KN/min; maximum load of 2000 N	Long axis coaxial to load direction	50
Pettersen 2009 [23]	MTS 858 Minibionix II, MTS Systems Corporation, Eden Prairie, MN, USA	6 LVDTs	SLS, SC	Abductor muscles, acetabulum	Vertical load 600 N (74 Kg BW); torsional moment 13.5 Nm (1.82%BW)	12° valgus tilt	50
Østbyhaug 2010 [22]	MTS 858 Minibionix II, MTS Systems Corporation, Eden Prairie, MN, USA	6 LVDTs	SLS, SC	Abductor muscles, acetabulum	Vertical load 600 N (74 Kg BW); torsional moment 15 Nm	12° valgus tilt	50
Wik 2011 [24]	MTS 858 MiniBionix II, MTS Systems Corporation, Eden Prairie, MN, USA	3 LVDTs	SLS, SC	Abductor muscles, acetabulum	Vertical force 600 N; torsional moment 13.8 Nm; for the strain measurement, torsional moment 10.0 Nm	12° valgus tilt	10S
Bieger 2012 [18]	Servo hydraulic machine, instron, Typ 8871, Pfungstadt, Germany	6 LVDTs	SLS	Acetabulum	1 Hz for first 4000 cycles; 0–1000 cycles 100–400 N; 1000–2000 cycles 100–800 N; 2000–3000 cycles 100–1200 N; 3000–4000 cycles 100–1600 N (250%BW); 2 Hz for 96,000 cycles with 100–1600 N	8° valgus, 6° flexion	250; 500
Bieger 2013 [19]	Instron, Typ 8871, Pfungstadt, Germany	6 LVDTs	SLS	NR	Vertical load 100–1600 N; frequency (2 Hz)	8° valgus, 6° flexion	500
Østbyhaug 2013 [15]	MTS 858 Minibionix II, MTS Systems Corporation, Eden Prairie, MN, USA	6 LVDTs	SLS, SC	Abductor muscles, acetabulum	Vertical load 600 N (73 Kg BW); torsional moment 15 Nm	12° valgus tilt	500
Bieger 2016 [13]	Instron, Typ 8871, Pfungstadt, Germany	6 LVDTs	SLS	NR	Vertical load 100–1600 N (250%BW); frequency (2 Hz)	8° valgus, 6° flexion	40 K, 100 K

*SLS* single-leg stance, *SC* stair climbing, *BW* body weight, *NR* not reported

**Table 3:** Composite femur and implant information of included studies with composite specimens

Author, year	Country	Composite femur Information		Subjects	Implant information			
		Characteristics	Femoral osteotomy		Stem	Coating	Characteristics	Femoral head
Viceconti 2000 [32]	Italy	Model 3103	NR	6	Anatomical cementless stem	NR	NR	NR
Viceconti 2001 [31]	Italy	Model 3103	NR	6	Anatomical cementless stem	NR	NR	NR
Heller 2005 [27]	Germany	Model 3103; size M	NR	6	CLS Spotorno stem	Full grit-blasted surface	Double-tapered design; straight collarless titanium stem; RCS; Riple-tapered collarless stem; size 11.25	NR
				6	Alloclassic SL stem	Grit-blasted surface	Straight, collarless; Distal anchorage concept with a predominantly meta-diaphyseal load transmission conical design	
Kassi 2005 [28]	Germany	Model 3103; size M	NR	6	CLS Spotorno stem	Full grit-blasted surface	Collarless; triple-tapered; titanium alloy (Ti6Al7Nb)	NR
Fottner 2009 [1, 26]	Germany	Model 3306; size L; left side; 3rd generation	20 cm below LT	5	TPP short stem	NR	Size 40	Standard ceramic head (32 mm, size M)
				5	Mayo short stem	Aluminum oxide roughed surface; Full grit-blasted surface	Neck preserving; a double-tapered design; RCS; CCD angle of 132°	
				6	Metha short stem	Proximal 2/3 coated with porous titanium and dicalcium phosphate; Polished distal	Partial neck preserving; anchored directly in the femoral neck and metaphysis; Size 3; CCD angle of 130° and 140°	
Fottner 2011 [2, 25]	Germany	Model 3406; size L; left side; 4th generation	25 cm below LT	6	CLS Spotorno stem	Full grit-blasted surface	Double-tapered design; straight collarless titanium stem; RCS	Standard ceramic head (32 mm, Size M)
Tuncay 2016 [30]	Turkey	Model 3403; size M; left side; 4th generation	NR	10	Cylindrical straight stems	NR	Metaphyseal and diaphyseal press-fit	+ 0 head; a cup similar to the acetabular liner
				10	RCSl, tapered stems (SL-Plus, no: 16; Smith & Nephew)	NR	Metaphyseal press-fit, tapered	
Fottner 2017 [14]	Germany	Model 3406; size L; left side; 4th generation	20 cm below LT	1	CLS Spotorno stem	Full grit-blasted surface	Double-tapered design; straight collarless titanium stem; RCS	Standard ceramic head (32 mm, size M)
Schmidutz 2017 [29]	Germany	Modell 3306; size S; left side	19.16 cm below LT	8	CLS Spotorno stem	Full grit-blasted surface	Double-tapered design; straight collarless titanium stem; RCS; CCD angle of 135°	Standard ceramic head (32 mm, size M)
		Model 3306; size M; left side	21.76 cm below LT	8				
		Model 3306; size L; left side	23 cm below LT	8				
Yan 2017 [33]	Germany	Model 3306; size L; left side	20 cm below LT	2	Metha short stem	Proximal 2/3 coated with porous titanium and dicalcium phosphate; Polished distal stem	Partial neck preserving; anchored within the femoral neck; double-tapered; collarless; CCD angle of 135°	Standard ceramic head (32 mm, size M)
				2	CLS Spotorno stem	Full-length grit-blasted surface	Double-tapered design; straight collarless titanium stem; rectangular cross-section; size 13.25; CCD angle of 135°	

*CCD* caput-collum diaphyseal, *L* large, *M* medium, *S* small, *LT* lesser trochanter, *NR* not reported

**Table 4:** Loading information of included studies with composite specimens

Author, year	Material testing machine	Detect Jig	Loading information			Test cycles
			Action simulated	Load force	Femur orientation	
Viceconti 2000 [32]	NR	5 LVDTs	SC	Vertical load 1700 N	NR	20
Viceconti 2001 [31]	NR	4 LVDTs	SC	Vertical load 1700 N	NR	20
Heller 2005 [27]	Dynamic testing machine (Instron 8871)	LVDTs	SC	2348 N; frequency (0.25 Hz)	10° adduction, 6° flexion	100
Kassi 2005 [28]	Dynamic servo-hydraulic testing machine (Instron 8871)	Force sensors, LVDTs	Walking, SLS, SC	Vertical load 1000 N; frequency (0.25 Hz); loaded with 50% and 75% of the computed peak loads, corresponding to a computed joint force of 1062 N/1174 N (walking/stair climbing) and 1593 N/1761 N	8° adduction, 1° flexion; 10° adduction, 6°flexion; only load a hip contact force, simulating stair climbing	100
Fottner 2009 [1, 26]	Hydraulic material testing device	6 LVDTs	Walking	Vertical load 100–1700 N (70 kg); frequency (1 Hz)	16° adduction, 9° posterior tilt	30
Fottner 2011 [2, 25]	Zwick/Z010	6 LVDT sensors	Walking	Vertical load 100–1700 N (70 kg); frequency (0.5 Hz)	16° adduction, 9° posterior tilt	30
Tuncay 2016 [30]	MTS 858 Mini Bionix II	NR	SLS	Vertical load 100–1000 N with a velocity of 50 N/s; 1000 N at 3 Hz for 10,000 cycles; torsional moment 0.5–10 Nm; frequency (3 Hz)	16° valgus tilt	10000
Fottner 2017 [14]	ElectroPuls E10000, Instron, Norwood, USA	6 LVDT sensors	Walking	Vertical load 300–1700 N (70 kg); frequency (1 Hz)	16° adduction, 9° posterior tilt	100
Schmidutz 2017 [29]	Hydraulic testing device	6 LVDT sensors**,** 6 strain gauges	Walking	Axial force of 250–1416.1 N (70 kg); frequency (1 Hz)	16° adduction, 9° posterior tilt	20
Yan 2017 [33]	Hydraulic testing device	6 LVDT sensors	Walking	Vertical load 100–1700 N (70 kg); frequency (1 Hz)	16° adduction, 9° posterior tilt	600

*SLS* single-leg stance, *SC* stair climbing, *NR* not reported

**Table 5:** Quality assessment of all included studies

Author, year	1	2	3	4	5	6	7	8	9	10
Gotze 2002 [20]	Y	Y	Y	Y/NA	N	Y	Y	Y	N	Y/Y
Abdul-Kadir 2008 [17]	Y	Y	Y	Y/Y	Y	Y	Y	Y	N	Y/Y
Bieger 2012 [18]	Y	Y	Y	Y/Y	Y	Y	Y	Y	Y	Y/Y
Bieger 2013 [19]	Y	Y	Y	Y/Y	Y	Y	Y	Y	N	Y/Y
Bieger 2016 [13]	Y	Y	Y	Y/Y	Y	Y	Y	Y	N	Y/Y
Klestil 2006 [21]	Y	Y	Y	Y/Y	N	Y	Y	Y	N	Y/Y
Østbyhaug 2010 [22]	Y	Y	Y	Y/NA	Y	Y	Y	Y	N	Y/Y
Østbyhaug 2013 [15]	Y	Y	Y	Y/NA	Y	Y	Y	Y	N	Y/Y
Pettersen 2009 [23]	Y	Y	Y	Y/NA	Y	Y	Y	Y	N	Y/Y
Wik 2011 [24]	Y	Y	Y	Y/NA	Y	N	Y	Y	N	Y/Y
Viceconti 2000 [32]	Y	Y	Y	Y/NA	N	Y	Y	Y	N	Y/Y
Viceconti 2001 [31]	Y	Y	Y	Y/NA	N	Y	Y	Y	N	Y/Y
Fottner 2009 [1, 26]	Y	Y	Y	Y/Y	Y	Y	Y	Y	N	Y/Y
Fottner 2011 [2, 25]	Y	Y	Y	Y/Y	Y	Y	Y	Y	N	Y/Y
Fottner 2017 [14]	Y	Y	Y	Y/Y	Y	Y	Y	Y	N	Y/Y
Heller 2005 [27]	Y	Y	Y	Y/Y	Y	Y	Y	Y	N	Y/Y
Kassi 2005 [28]	Y	Y	Y	Y/Y	Y	Y	Y	Y	N	Y/Y
Schmidutz 2017 [29]	Y	Y	Y	Y/Y	Y	Y	Y	Y	N	Y/Y
Tuncay 2016 [30]	Y	Y	N	Y/Y	N	Y	Y	Y	N	Y/Y
Yan 2017 [33]	Y	Y	Y	Y/Y	Y	Y	Y	Y	N	Y/Y

Criteria 1, aims description; 2, dependent variables description; 3, interventions description; 4, load/speed order randomized; 5, setting description; 6, date collection description; 7, data analysis description; 8, statistical description; 9, drop outs; 10, point estimate/variability

### Evidence network

**Fig. 3:**
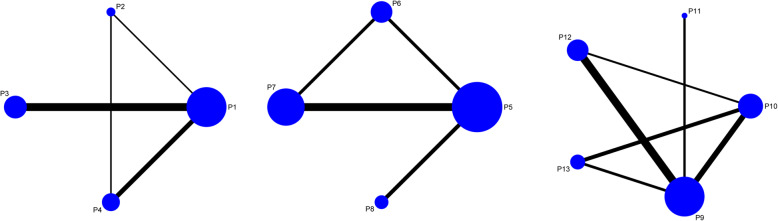
Network evidence for the comparison of micromotions in four directions at the proximal, middle, and distal portion of the femoral stem

As shown in Fig. 3, the lines between two connected points represent direct comparison. Points without connection indicate comparisons indirectly through the network meta-analysis. The width of the lines represents the number of sets of data from included studies, whereas the size of the nodes demonstrates the overall sample size of P1 to P13.

### Forest plots of network meta-analyses

**Fig. 4:**
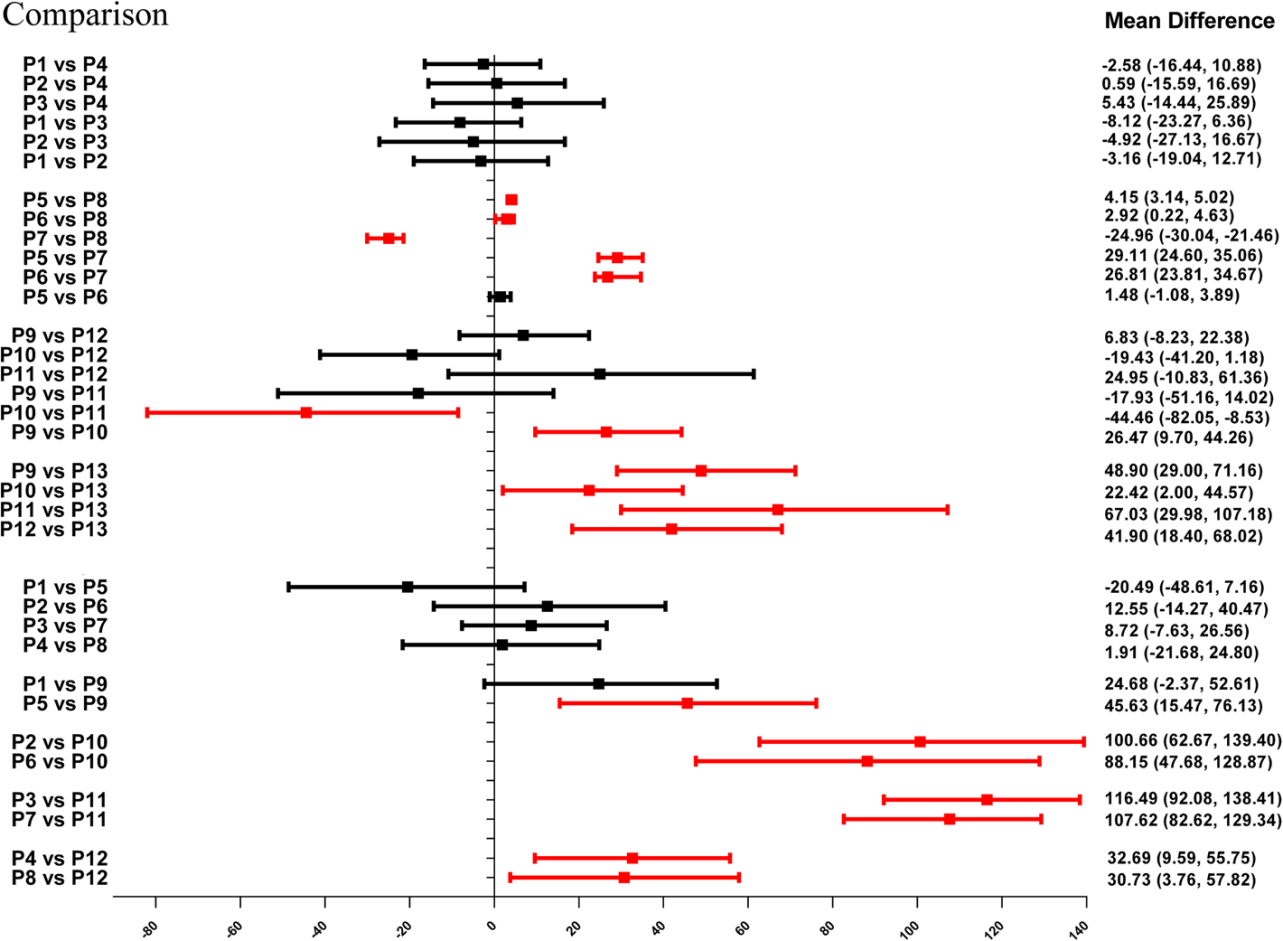
Forest plots for the comparisons of micromotions between the femoral stem and bone in four directions at the horizontal (proximal, middle, and distal) and the vertical level (anterior, posterior, medial, and lateral)

As indicated in Fig. 4 and Table 6, P13 ranked the highest micromotion, which means it is most likely to occur aseptic loosening. In the horizontal level, the arrangements of micromotions between femoral stem and bone using MDs for the comparisons at the proximal, middle, and distal part was P1 = P2 = P3 = P4, P7 > P8 > P6 = P5 and P10 ≥ P12 = P9 = P11, respectively. In the vertical level, the arrangements of micromotions between femoral stem and bone for the comparisons involving the anterior, posterior, medial, and lateral directions was P9 > P5 = P1, P10 > P6 > P2, P11 > P7 > P3, P12 > P8 > P4, respectively, which demonstrated that the micromotions in the distal part of femoral stem is higher than that in the medial and proximal part, while the micromotions in the medial part of femoral stem is higher than that in the proximal part. Contribution plot of network meta-analysis shows the contribution of each direct and indirect comparison to the network summary effects in the Appendix 2.

**Table 6:** The results of network meta-analysis

Vertical				
**Anterior**				
**P1**	− 20.49 (− 48.61, 7.16)	24.68 (− 2.37, 52.61)		
20.49 (− 7.16, 48.61)	**P5**	**45.63 (15.47, 76.13)**		
− 24.68 (− 52.61, 2.37)	**− 45.63 (− 76.13, − 15.47)**	**P9**		
**Posterior**				
**P2**	12.55 (− 14.27, 40.47)	**100.66 (62.67, 139.40)**		
− 12.55 (− 40.47, 14.27)	**P6**	**88.15 (47.68, 128.87)**		
**− 100.66 (− 139.40, − 62.67)**	**− 88.15 (− 128.87, − 47.68)**	**P10**		
**Medial**				
**P3**	8.72 (− 7.63, 26.56)	**116.49 (92.08, 138.41)**		
− 8.72 (− 26.56, 7.63)	**P7**	**107.62 (82.62, 129.34)**		
**− 116.49 (− 138.41, − 92.08)**	**− 107.62 (− 129.34, − 82.62)**	**P11**		
**Lateral**				
**P4**	1.91 (− 21.68, 24.80)	**32.69 (9.59, 55.75)**		
− 1.91 (− 24.80, 21.68)	**P8**	**30.73 (3.76, 57.82)**		
**− 32.69 (− 55.75, − 9.59)**	**− 30.73 (− 57.82, − 3.76)**	**P12**		
**Horizontal**				
**Proximal**				
**P1**	− 3.16 (− 19.04, 12.71)	− 8.12 (− 23.27, 6.36)	− 2.58 (− 16.44, 10.88)	
3.16 (− 12.71, 19.04)	**P2**	− 4.92 (− 27.13, 16.67)	0.59 (− 15.59, 16.69)	
8.12 (− 6.36, 23.27)	4.92 (− 16.60, 27.13)	**P3**	5.43 (− 14.44, 25.89)	
2.58 (− 10.88, 16.44)	− 0.59 (− 16.69, 15.59)	− 5.43 (− 25.89, 14.44)	**P4**	
**Middle**				
**P5**	1.48 (− 1.08, 3.89)	**29.11 (24.60, 35.06)**	**4.15 (3.14, 5.02)**	
− 1.48 (− 3.89, 1.08)	**P6**	**26.81 (23.81, 34.67)**	**2.92 (0.22, 4.63)**	
**− 29.11 (− 35.06, − 24.60)**	**− 26.81 (− 34.67, − 23.81)**	**P7**	**− 24.96 (− 30.04, − 21.46)**	
**− 4.15 (− 5.02, − 3.14)**	**− 2.92 (− 4.63, − 0.22)**	**24.96 (21.46, 30.04)**	**P8**	
**Distal**				
**P9**	**26.47 (9.70, 44.26)**	− 17.93 (− 51.16, 14.02)	6.83 (− 8.23, 22.38)	**48.90 (29.00, 71.16)**
**− 26.47 (− 44.26, − 9.70)**	**P10**	**− 44.46 (− 82.05, − 8.53)**	− 19.43 (− 41.20, 1.18)	**22.42 (2.00, 44.57)**
17.93 (− 14.02, 51.16)	**44.46 (8.53, 82.05)**	**P11**	24.95 (− 10.83, 61.36)	**67.03 (29.98, 107.18)**
− 6.83 (− 22.38, 8.23)	19.43 (− 1.18, 41.20)	− 24.95 (− 61.36, 10.83)	**P12**	**41.90 (18.40, 68.02)**
**− 48.90 (− 71.16, − 29.00)**	**− 22.42 (− 44.57, − 2.00)**	**− 67.03 (− 107.18, − 29.98)**	**− 41.90 (− 68.02, − 18.40)**	**P13**

### Inconsistency test of network meta-analyses

**Fig. 5:**
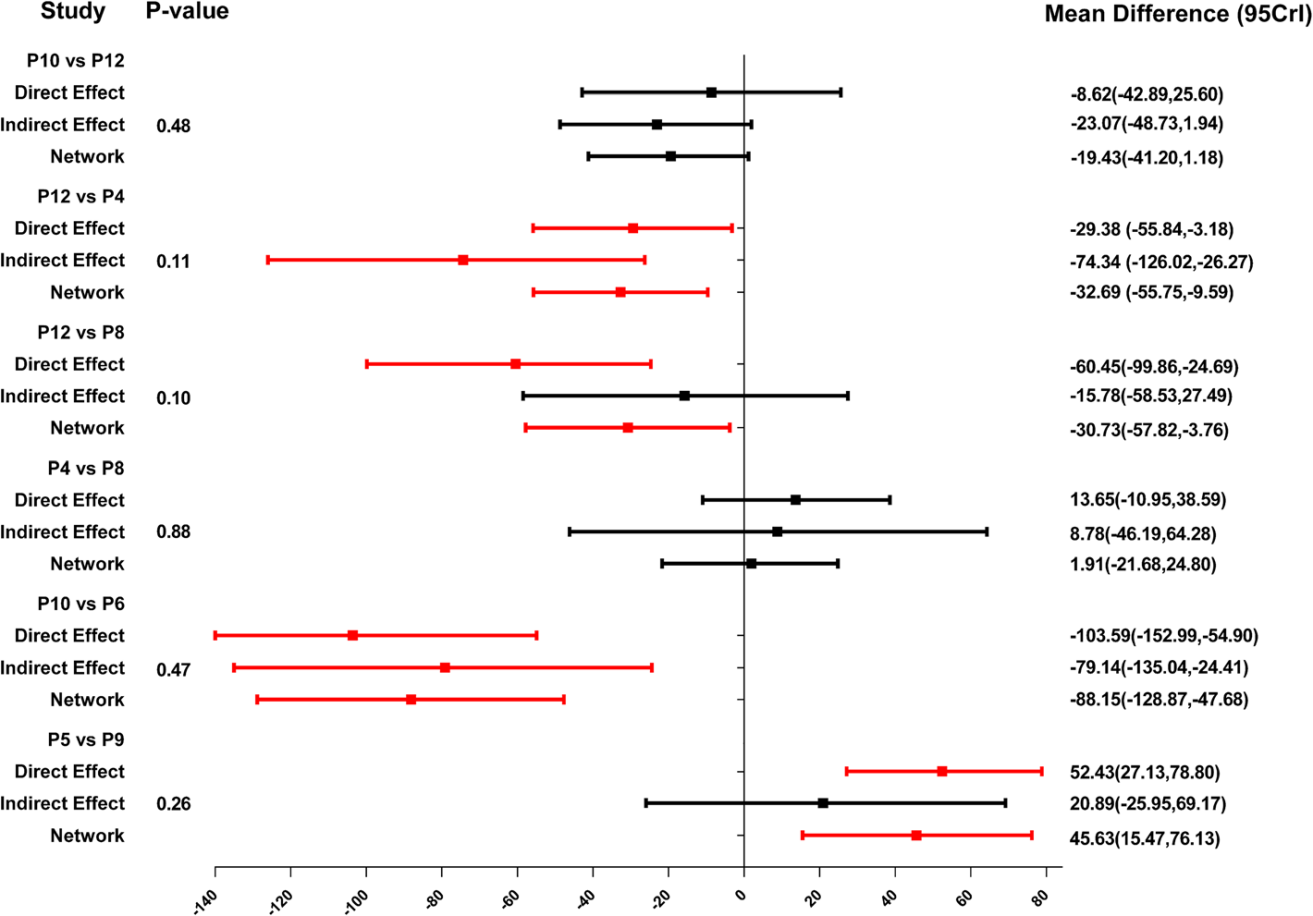
Inconsistency test for direct and indirect comparisons of micromotions at the proximal, middle, and distal portion of the femoral stem

The node-splitting method and its Bayesian *P* value used to report the inconsistency of our results showed that the confidential intervals in all loops were crossed over with blank value (*P* > 0.05), and direct and indirect estimates of the effects in network meta-analysis were not significantly different, with good homogeneity (Fig. 5). And then sensitivity analysis was performed to compare the analysis result of the random-effects model ([Dbar] = 49.48, [pD] = 32.37, [DIC] = 81.85, and *I*^2^ = 0) and fixed-effect model ([Dbar] = 56.92, [pD] = 26.00, [DIC] = 82.92, and *I*^2^ = 3%), which demonstrated that the results of two models were similar; therefore, the results were steady.

### The SUCRA of micromotions of 13 measurement points

**Fig. 6:**
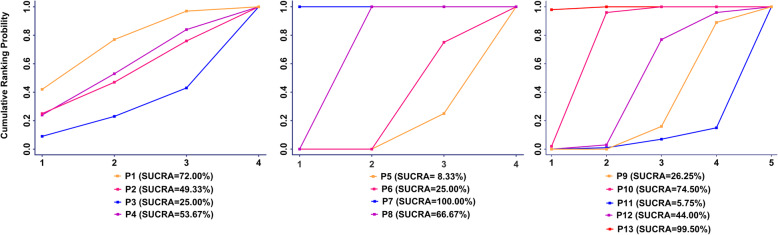
Rank probability for the comparisons of micromotions between femoral stem and bone in the horizontal level (proximal, middle, and distal)

**Fig. 7:**
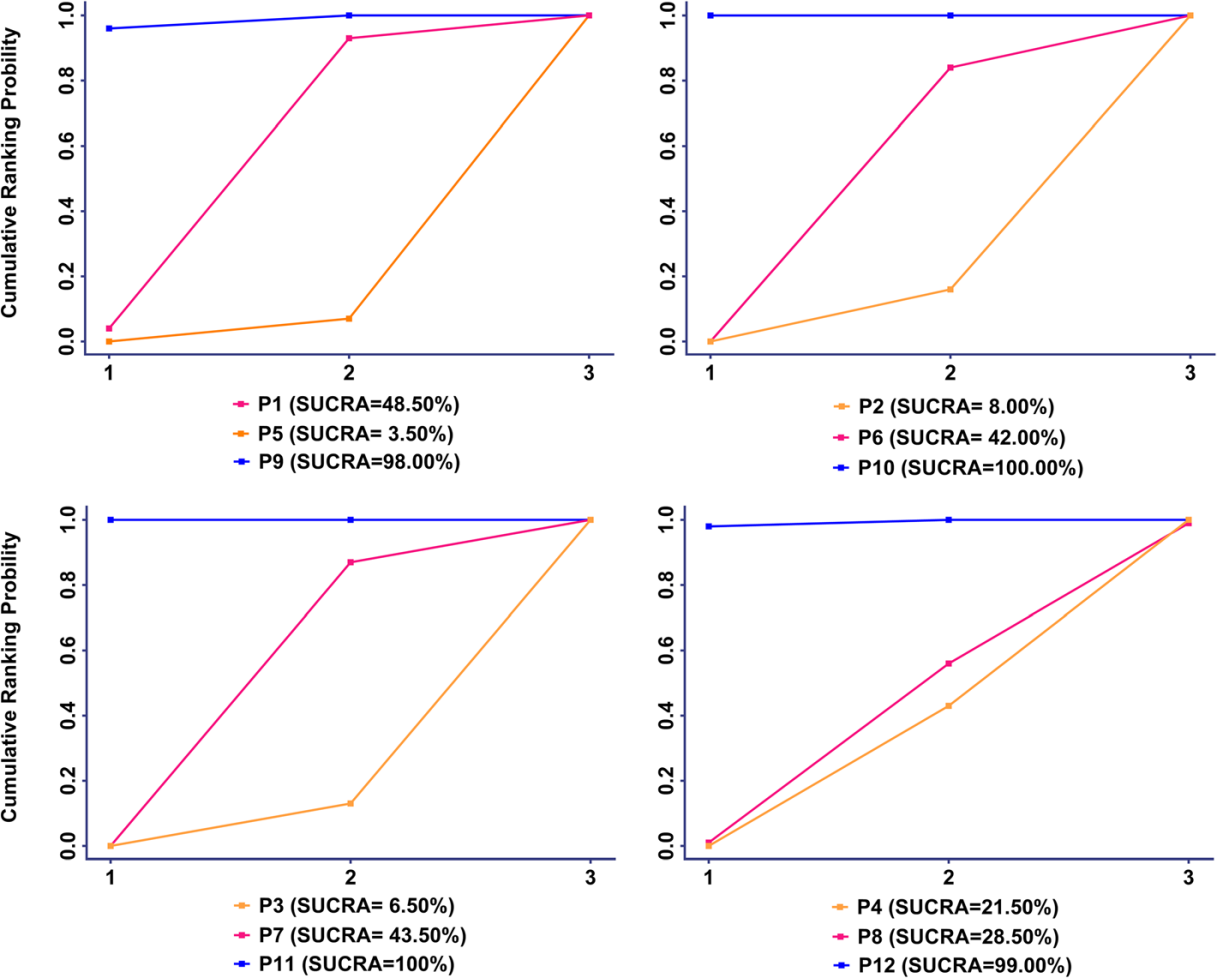
Rank probability for the comparisons of micromotions between femoral stem and bone in the vertical level (anterior, posterior, medial, and lateral)

As indicated in Figs. 6 and 7, P13 ranked the highest micromotion, which means it is most likely to occur aseptic loosening. In the horizontal level, the arrangement of rank probability for the comparisons of micromotions between femoral stem and bone in four directions at the proximal, middle, and distal part was P1 > P4 = P2 > P3, P7 > P8 > P6 > P5 and P10 > P12 > P9 > P11, respectively (Fig. 6). In the vertical level, the arrangement of rank probability for the comparisons of anterior, posterior, medial, and lateral micromotions between femoral stem and bone was P9 > P1 > P5, P10 > P6 > P2, P11 > P7 > P3 and P12 > P8 > P4, respectively (Fig. 7).

### Publication bias

**Fig. 8:**
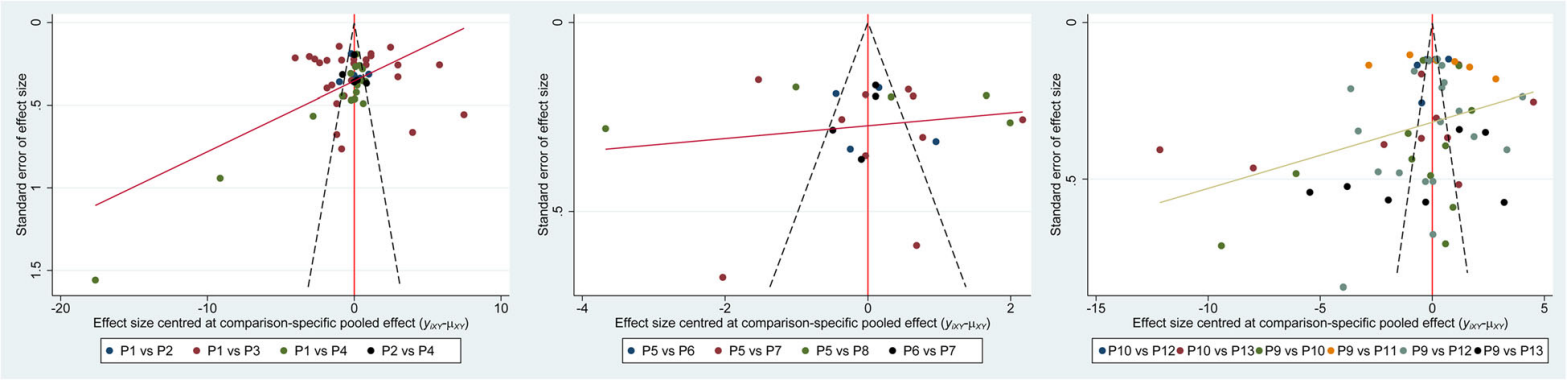
Publication bias of the comparison for micromotions of four directions in the proximal, middle, and distal portion of femoral stem

Figure 8 implied that no evident publication bias of the comparison for micromotions of four directions in the proximal, middle, and distal portion of femoral stem surface was observed in this network meta-analysis. However, funnel plots provide only hints of publication bias and not definite proof; therefore, the results should be interpreted cautiously.

## Discussion

It is a well-known fact that infectious loosening and aseptic loosening are the problems of artificial joint in clinical which needs to be addressed. This network meta-analysis provides evidence-based principles from the biomechanical rationale that micromotions between femoral stem and bone in four directions is similar at the proximal, P7 is the highest point at the middle part, and P10 is the highest point at the distal part. P13 is the highest point of micromotions at implant bone surface, while the results demonstrated that the micromotions in the distal part of the femoral stem seem to be higher than that in the middle and proximal part. Additionally, the micromotions in the medial part of femoral stem seem to be higher than that in the proximal part.

The biomechanical method for measurement of micromotions is very accurate due to a resolution of 0.1 μm but also a very complex procedure. The accurate measurement lies within the 3D measurement. However, most studies examined relative micromotion of every measurement point at implant bone surface utilizing only one LVDT [16, 28, 41], which usually only measured the spatial dimension of the movement orthogonal to the bone in most cases. At such, comparing published results of micromotion measurements at implant bone surface is challenging due to multiple factors such as different test set-ups, loading conditions, and specimen used and may bring errors for the final results. Fortunately, numerous studies have tried to figure out these factors. Previous biomechanical studies demonstrated that undersizing of cementless hip stems is a risk factor for aseptic loosening and early subsidence than appropriate size [25, 26]. Meanwhile, composite femur size and different offset versions (increased femoral offset and altered neck version) of cementless hip prostheses seem to be a minor influence factor for the primary stability [18, 24–26, 29]. Micromotions may be underestimated and the primary stability overestimated without a special emphasis on active simulation of muscle forces [28]. A biomechanical analysis performed by Heller et al. presented that the anchorage concept of cementless stems had a significant influence on the initial interface micromotions [27]. Two biomechanical studies presented that loading cycles (40,000 vs*.*100,000 and 3000 vs*.* 8000) seem to be a minor influence factor for the measurement of micromotion [13, 19]. Another biomechanical study suggested that stair climbing of patient activity induced the highest mechanical instability at the bone-prosthesis interface, which may compromise the necessary osseointegration process [28]. There were no significant differences in the anterior, lateral, or posterior interface cyclical motions for any of the stem shortening levels in neither one leg stance nor stair climbing [15]. Nevertheless, with the chosen position of the leg with adduction as well as flexion, a reasonable amount of torsional moment is applied, taking to some extent torsional moments into account that occur during stair climbing [42].

As specimens for the measurement of micromotions, fresh frozen human cadaveric femurs with the more similar biomechanical characteristics and anatomical structures to human and synthetic composite femurs with the advantages of consistent geometry and mechanical properties are commonly used. According to our results, a total of ten studies [14, 25–33] that chose the composite femurs and 10 studies [13, 15, 17–24] that chose the cadaver femurs demonstrated that femur difference did not notably influence their micromotions.

In this network meta-analysis, the highest 3D micromotion is registered at the distal tip of the femoral stem, which is in accordance with the previous biomechanical studies [14, 18, 27, 30]. Three studies [25, 29, 33] demonstrated that the highest micromotion of the femoral stem at the distal tip reached or exceeded the threshold for osseous integration of 150 μm, which was related to the design rationale with a proximal anchorage and load transfer.

This network meta-analysis provided evidence-based principles from the biomechanical rational that micromotions between femoral stem and bone in four directions was similar in the proximal part while medial point and posterior point registered the highest micromotions in the middle and distal part, respectively, while the results demonstrated that the micromotions in the distal part of the femoral stem seem to be higher than that in the middle and proximal part. Even though we could not detect significant different results in respect to comparison of P9 vs. P5, there was a tendency towards higher micromotion registers at P9 compared to the P5. Additionally, the micromotions in the medial part of femoral stem seem to be higher than that in the proximal part.

The concept of cementless fixation can be addressed with different design concepts [10], which at a certain presented the locking sites, such as the Zweymüller-type prosthesis locking in the distal metaphysis and proximal diaphysis with a four-point fixation concept and the CLS Spotorno stem involving the stem designs of the proximal anchoring [10, 43]. These two kinds of stems exhibited similar movement patterns moved mainly in the distal direction. Relatively small interface movements were registered in the antero-posterior direction. These results reflect the stem design with an intended proximal load transfer and a thin distal part of the stem not filing the intramedullary canal to prevent load transmission in this area [44, 45]. Distal locking mechanism of the Zweymüller stem showed proximal bone atrophy as a result of stress shielding in vivo [46]. The thin distal stem is not intended to fit and fill the medullary canal in order to avoid stress shielding [22] and also does not need osseous integration to result in a secondary stable stem [12]. 3D micromotion of CLS stem at the distal tip reached the design rationale with a proximal anchorage and load transfer. In contrast, the 3D micromotions of the remaining measurement points 1 to 4 were clear below 100 μm in all examined sizes. This finding indicates that osseous integration is to be expected from the level of the lesser trochanter till the middle of the stem.

There are still some limitations even through the rigorous analyses that were conducted. First of all, methodological variability and different loading scenarios along with varying measurement devices and locations, and comparisons of the experimental outcome of different research laboratories are very difficult. Besides different sizes of cadaver and composite femurs and various types of prostheses, the evaluation and comparison of micromotions varied in these biomechanics meaning that heterogeneity was unavoidable even though subgroup and sensitivities analyses were performed with rigorous methods. Second, some characteristics of the femurs and prostheses, such as simulated action, anatomical features simulated, femoral osteotomy, and test cycles were not provided by the literature. Third, several subgroup analyses were based on a small number of studies or were impossible because of incomplete data, which influence statistical algorithms and their deductions. Finally, the present study only enrolled published literature. Some unpublished ones which met inclusion criteria might be missed and publication bias was shown in some subgroup analyses.

## Conclusions

The network meta-analysis providing evidence-based principles from the biomechanical rationale for micromotions between femoral stem and bone seems to reveal that the distal part of the femoral stem is easier to register higher micromotion and the tip point of the femoral stem registers the highest micromotions.

## Supplementary information

**Additional file 1: Appendix Table 1.** Included studies, with website links enabling direct access to each corresponding article abstract. **Appendix figure 1.** Contribution plot for the comparison of micromotions in four directions at the proximal, middle and distal portion of femoral stem surface.

## Data Availability

All data generated or analyzed during this study are included in this published article.

## Abbreviations

CI: Confidence interval
MD: Mean difference
PRISMA: Preferred Reporting Items for Systematic Reviews and Meta-Analyses
STROBE: Strengthening the Reporting of Observational studies in Epidemiology
SUCRA: Surface under the cumulative ranking curve
THA: Total hip arthroplasty
