# Supplementary material for: Comparisons of the surface micromotions of cementless femoral prosthesis in the horizontal and vertical levels: a network analysis of biomechanical studies
Source: J Orthop Surg Res. 2020 Jul 31;15:293. doi: 10.1186/s13018-020-01794-4 (PMC7393913; doi:10.1186/s13018-020-01794-4)
Supplement: Supplementary file 1 — Additional file 1: Appendix Table 1. Included studies, with website links enabling direct access to each corresponding article abstract. Appendix figure 1. Contribution plot for the comparison of micromotions in four directions at the proximal, middle and distal portion of femoral stem surface. [file 13018_2020_1794_MOESM1_ESM.docx]

| **Study, year** | **website links enabling direct access**  **to the abstract of included studies** |
| --- | --- |
| Gotze 2002 | https://www.ncbi.nlm.nih.gov/pubmed/12034119 |
| Klestil 2006 | https://www.ncbi.nlm.nih.gov/pubmed/16557367 |
| Abdul-Kadir 2008 | https://www.ncbi.nlm.nih.gov/pubmed/18036531 |
| Pettersen 2009 | <https://www.ncbi.nlm.nih.gov/pubmed/19368993> |
| Østbyhaug 2010 | https://www.ncbi.nlm.nih.gov/pubmed/20129726 |
| Wik 2011 | https://www.ncbi.nlm.nih.gov/pubmed/21922956 |
| Bieger 2012 | <https://www.ncbi.nlm.nih.gov/pubmed/21889243> |
| Bieger 2013 | <https://www.ncbi.nlm.nih.gov/pubmed/23553802> |
| Østbyhaug 2013 | <https://www.ncbi.nlm.nih.gov/pubmed/23637258> |
| Bieger 2016 | <https://www.ncbi.nlm.nih.gov/pubmed/27380777> |
| Viceconti 2000 | <https://www.ncbi.nlm.nih.gov/pubmed/11006385> |
| Viceconti 2001 | <https://www.ncbi.nlm.nih.gov/pubmed/11714554> |
| Heller 2005 | <https://www.ncbi.nlm.nih.gov/pubmed/16429942> |
| Kassi 2005 | <https://www.ncbi.nlm.nih.gov/pubmed/15797595> |
| Fottner 2009 | <https://www.ncbi.nlm.nih.gov/pubmed/19307048> |
| Fottner 2011 | <https://www.ncbi.nlm.nih.gov/pubmed/21536357> |
| Tuncay 2016 | <https://www.ncbi.nlm.nih.gov/pubmed/26795256> |
| Fottner 2017 | <https://www.ncbi.nlm.nih.gov/pubmed/28865042> |
| Schmidutz 2017 | <https://www.ncbi.nlm.nih.gov/pubmed/27960138> |
| Yan 2017 | <https://www.ncbi.nlm.nih.gov/pubmed/28488162> |

**Appendix Table 1.** Website links enabling direct access to the abstract of included studies.


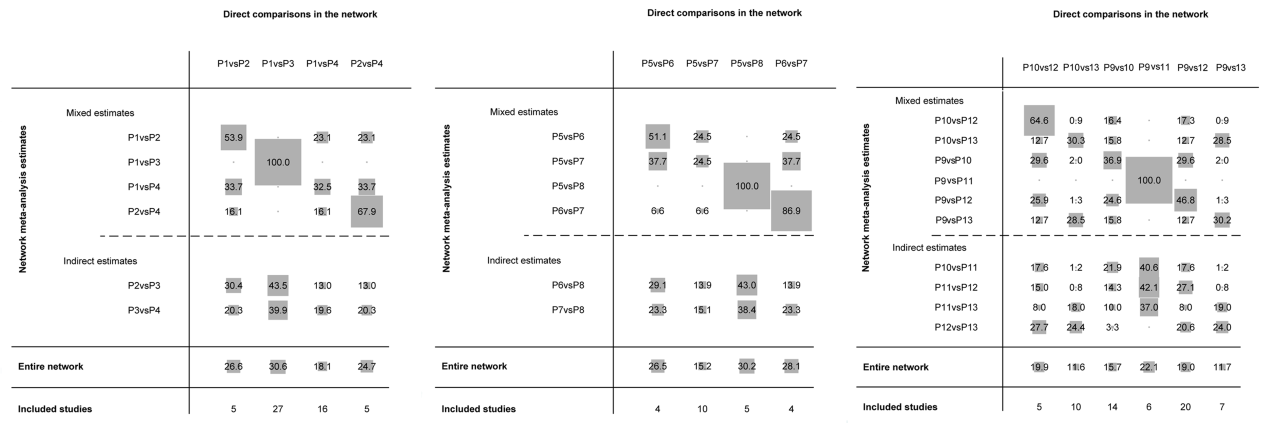


**Appendix Figure 1.** Contribution plot for the comparison of micromotions in four directions at the proximal, middle and distal portion of femoral stem surface.
